# Brainsourcing for temporal visual attention estimation

**DOI:** 10.1007/s13534-024-00449-1

**Published:** 2025-01-11

**Authors:** Yoelvis Moreno-Alcayde, Tuukka Ruotsalo, Luis A. Leiva, V. Javier Traver

**Affiliations:** 1 Institute of New Imaging Technologies, Universitat Jaume I, Castellón de la Plana, Spain; 2 University of Copenhagen, Copenhagen, Denmark; 3 LUT University, Lahti, Finland; 4 University of Luxembourg, Esch-sur-Alzette, Luxembourg

**Keywords:** Brain-computer interfacing, EEG, Visual attention, Brainsourcing

## Abstract

The concept of *temporal* visual attention in dynamic contents, such as videos, has been much less studied than its *spatial* counterpart, i.e., visual salience. Yet, temporal visual attention is useful for many downstream tasks, such as video compression and summarisation, or monitoring users’ engagement with visual information. Previous work has considered quantifying a temporal salience score from spatio-temporal user agreements from gaze data. Instead of gaze-based or content-based approaches, we explore to what extent only brain signals can reveal temporal visual attention. We propose methods for (1) computing a temporal *visual* salience score from salience maps of video frames; (2) quantifying the temporal *brain* salience score as a cognitive consistency score from the brain signals from multiple observers; and (3) assessing the correlation between both temporal salience scores, and computing its relevance. Two public EEG datasets (DEAP and MAHNOB) are used for experimental validation. Relevant correlations between temporal visual attention and EEG-based inter-subject consistency were found, as compared with a random baseline. In particular, effect sizes, measured with Cohen’s *d*, ranged from very small to large in one dataset, and from medium to very large in another dataset. Brain consistency among subjects watching videos unveils temporal visual attention cues. This has relevant practical implications for analysing attention for visual design in human-computer interaction, in the medical domain, and in brain-computer interfaces at large.

## Introduction

Visual attention is a key cognitive process for humans to interact with their physical and digital environments. Understanding visual attention is important, among others, in the context of Human-Computer Interaction (HCI) and in Medical settings. In HCI, visual attention can inform the design of user interfaces and systems that align with how humans process visual information [1]. By understanding how users allocate their attention, designers can create more intuitive, efficient, and user-friendly interfaces, optimise information presentation, and enhance user engagement and experience [2, 3]. In the medical domain, visual salience can help in diagnosing abnormalities [4], or understanding how medical staff process biological images [5], which can provide training support to novel physicians. While behavioural and physiological signals have been exploited for salience estimation [6, 7], electroencephalography (EEG) signals are one of the most important as they carry information measured directly from the brain and have been applied in Brain-Computer Interfaces (BCI), such as virtual reality [8] or video event monitoring [9].

Previous work has explored the connection of brain signals with visual attention. Early research focused on simple tasks based on “search arrays” stimuli, containing many items, one of which would appear as highly visually salient [10]. While a large body of experiments has been mainly concerned with simple tasks [11, 12], there is mounting evidence that perceptual salience is not a fixed quantity, but rather is strongly modulated in real-time by the task demands [13]. For example, the P300 component, associated to visuo-spatial attention of event-related potential (ERP) correlates with the presentation of visual stimuli using EEG [14].

Recently, it has been shown that machine learning can predict attention during mental arithmetic tasks from EEG signals [15]. The decrease in the $\alpha$ frequency band and the increase in the $\beta$ band were reported to be markers of visual spatial attention [16]. A link between the $\beta$ band and visual attention has been shown [17], although recent findings [18] suggest that inter-frequency bands coupling may support high-level cognition functions such as affect [19]. The relationship of neural activity with visual attention was studied with simultaneous EEG-fMRI devices [20]. Some work has explored how to estimate visual salience maps from EEG [21]. Temporal attention was found to modulate the low-band alpha rhythm [22] and a quantification method of visual attention based on head movements using an eye-tracker has been proposed [23].

In this work, we explore whether the inter-subject brain responses consistency (a measure of EEG signal agreement) may reveal the temporal visual attention of the video stimuli. To that end, the *visual* attention is quantified from the compactness of the salience maps (i.e. how small and close together are the predicted salient image regions), and the *brain* consistency is estimated from the features computed from the EEG signals recorded from multiple observers while they watched the corresponding videos. Since the approach involves the brain signals from several users, we refer to this as this *brainsourcing* (after “brain” and “crowdsourcing”), as proposed previously in other contexts [24].

Our work differs from previous work in several respects. On the one hand, the stimuli we consider (videos) are more general than the ones considered in experiments conducted in vision science, which tend to use specific tasks that are performed under highly controlled conditions. In contrast, we consider task-free viewing conditions that do not artificially prime the participants to attend to a specific features of visual stimuli. On the other hand, computational models mostly focus on spatial salience, ignoring the *temporal* dimension of attention, whereas we define and propose novel ways of quantifying temporally both the visual salience and the brain consistency.

**Fig. 1:**
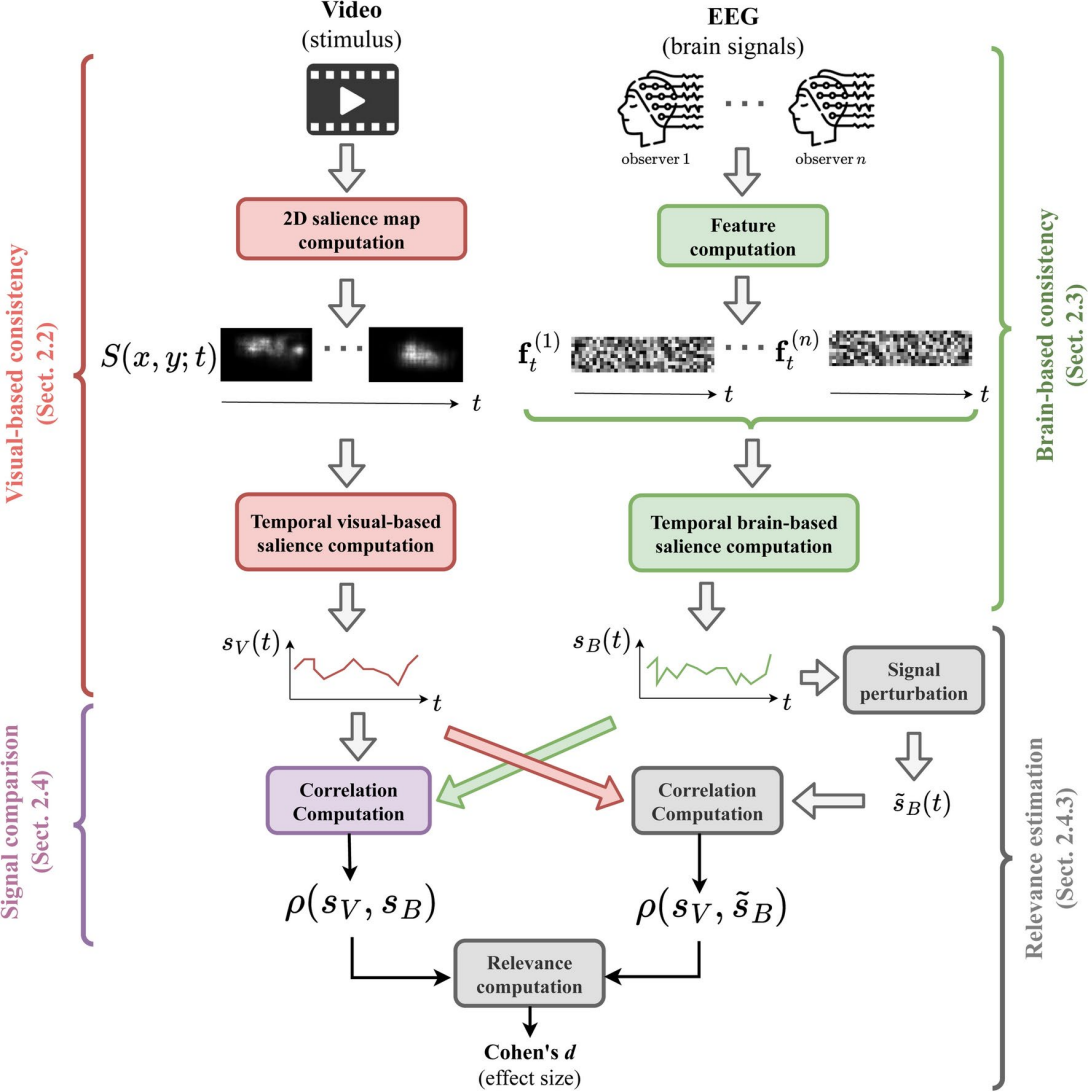
Overview of our approach. The upper-left part (red): salience maps $S(x,y;t)$ are computed from input video frames; then the temporal visual salience score $s_V(t)$ is computed (Sect. 2.2). The upper-right part (green): from the EEG signals of multiple observers of the video, brain features $\textbf{f}_t^{(i)}$ are extracted for every observer *i*, and from the set of these EEG features the brain-based temporal salience score $s_B(t)$ is then computed (Sect. 2.3). Middle/lower-left part (violet): the correlation between the signals $s_V(t)$ and $s_B(t)$ is computed. The lower-right and bottom parts (gray): the correlation between the original $s_V$ and a perturbed version of $s_B$, $\tilde{s}_B$, is computed (Sect. 2.4), and then compared with the correlation between the original signals, to estimate its relevance (Sect. 2.4.3), quantified as an effect size. (color figure online)

## Methodology

Our methodology relies on inter-subject brain response consistency and the compactness of the salience maps. Both concepts are inspired by the gaze-based spatio-temporal multi-observer agreement measure (Sect. 2.1). Then we propose the procedure to quantify the temporal *visual*-based salience score from video contents (Sect. 2.2). Next, the quantification of the temporal *brain*-based salience score from the EEG signals is described (Sect. 2.3). Finally, a local-based metric to quantify the correlation between both temporal signals, and the relevance of such correlation, are discussed (Sect. 2.4). An overview of the full procedure is illustrated in Fig. 1.

As a terminology clarification, we use *consistency* to refer to the underlying concept that is used in the input signals to compute the *temporal salience score* as the resulting output. Note also that we rely on visual salience maps to define a temporal salience score which can be understood as a scalar measure of temporal visual attention. Since modern learning-based methods for salience map estimation use human fixation points as ground-truth data, they have the potential to model human gaze behaviours which, in turn, are guided not only by low-level bottom-up visual cues as in the earliest salience models [25], but also by higher-level semantic concepts such as meaning [26].

### Gaze-based consistency

GLIMPSE [7], a gaze-based consistency measure for video contents, relies on the gaze points from a set of observers. Let $\textbf{g}(o,t)$ the gaze coordinates of video observer *o* at time *t*, and $\mathcal {P}_t$ the set of *n* gaze points from the *n* observers within a temporal window of length $2\theta _t+ 1$ centred at *t*, i.e.

1 $$\begin{aligned} \mathcal {P}_t = \Big \{\textbf{g}(o,t) : o \in \{1,\ldots ,N\}, \ t \in \left[ t-\theta _t,t+\theta _t\right] \Big \}. \end{aligned}$$

The measure of gaze-based temporal salience $s_G(t)$ is computed on normalised gaze coordinates as

2 $$\begin{aligned} s_G(t) = \frac{\sum _{\begin{array}{c} i,j\in \{1,\ldots ,n\} \\ i\ne j \end{array}} \mathbbm {1}[d_{ij} < \theta _s]}{\frac{n (n-1)}{2}} , \quad t\in \{1,\ldots ,T\}, \end{aligned}$$

with $d_{ij}$ being the pairwise Euclidean distance between the $i^{th}$ and $j^{th}$ points in the set $\mathcal {P}_t$, and $\mathbbm {1}[p]$ the indicator function. This score correlates well with notable dynamic events in the video contents [7]. Two scale parameters (temporal $\theta _t$ and spatial $\theta _s$) are involved in Eqs. 1 and 2. The temporal scale $\theta _t$ has a smoothing effect, and the spatial scale represents a sensitivity threshold. The subscript *G* in $s_G(t)$ is meant to represent the *gaze*-based temporal salience score, so that it can be easily distinguished this from the two other versions of the temporal salience score (visual-based and brain-based) considered below.

### Visual consistency

Visual salience maps encode the prediction of image areas that most likely attract the viewers attention and are a proxy for human visual attention [27]. Intuitively, how much these areas spread within the image allows us to quantify the consistency: less spread relates to higher consistency, and vice versa. In the case of a digital video, the consistency of a particular video frame at time *t* corresponds to the *temporal* visual attention score at that time *t*.

For the quantification of this consistency, the GLIMPSE concept is adapted from the discrete set of gaze points to the 2D image plane with real values. Given a 2D salience map at a particular time *t*, $S(x,y;t)\in [0,1]$ of size $w\times h$, the key idea is to interpret $S(x,y)$ as the gaze probability or, in other words, the ratio of the number observers who gazed over $(x,y)$ over the total number of hypothetical observers.

We derived and define the visual-based temporal salience score at each frame as

3 $$\begin{aligned} s_V = \frac{\sum _{i=1}^m \sum _{j=1}^m S(x_i,y_i)\cdot S(x_j,y_j) \cdot \mathbbm {1}[d_{ij}<\theta _s]}{\sum _{i=1}^m \sum _{j=1}^m S(x_i,y_i)\cdot S(x_j,y_j)}, \end{aligned}$$

where $m = w \cdot h$ is the number of pixels of the salience map. Note that Eq. 3 lends itself to an intuitive explanation: the ratio of image location pairs, weighted by their underlying gaze probabilities, whose distance is below a distance threshold $\theta _s$, with respect to all possible pairs. Therefore, it can be seen in essence as the probabilistic real-valued and 2D grid-based counterpart of the integer-valued and set-based nature of $s_G$. 

For salience map computation, the computational model TASED [28] was used. We run the authors-provided pre-trained version^1^ on the video frames, resulting in $w\times h = 224\times 384$ salience maps *S*, whose values were normalised to [0, 1] by dividing by the maximum value, $S(x,y)/\max _{x,y} \{S(x,y)\}$.

The threshold $\theta _s$ for $s_V$ in Eq. 3 was heuristically set to $10\%$ of the arithmetic mean of the salience map dimensions *w* and *h*, somehow following the criteria for GLIMPSE, which suggested $\theta _s=0.1$ for gaze coordinates normalised to [0, 1].

For details for an efficient implementation of Eq. 3, please refer to Appendix A. Note that, for simplicity, the temporal index *t* is dropped from $s_V(t)$ and $S(x,y;t)$. Also note that, unlike the temporal and scale parameter in the gaze-based measure (Sect. 2.1), the proposed salience-based measure (Eq. 3) only considers the spatial one since the effect of the temporal parameter is simply a temporal smoothing [7], which can be captured by either averaging the salience maps in a temporal window before computing Eq. 3 or by smoothing the resulting temporal signal $s_V(t)$. In this work, we perform none of these averaging choices, since the salience maps are already computed from a set of neighbouring video frames and therefore implicitly include this temporal effect.

**Fig. 2:**
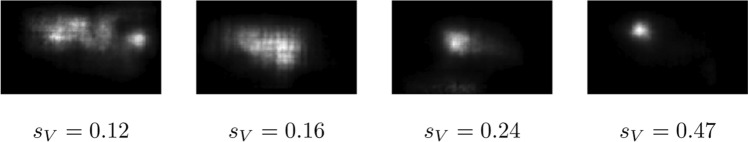
Visual salience-based score $s_V$ for salience maps of video frames from DEAP dataset. It can be noticed how the score increases from left to right, from more scattered to more concentrated salience maps $S(x,y;t)$

Examples of values of $s_V(t)$ for salience maps $S(x,y;t)$ computed with TASED on videos from the DEAP dataset (introduced in Sect. 3.1) are given in Fig. 2. It can be observed how the visual-based score $s_V$ captures the spread of the values in the salience map, as motivated at the beginning of this section.

### Brain signal inter-subject agreement

To compute a measure of consistency for brain signals (inter-observer agreement), a feature vector $\textbf{f}_t$ of the EEG signal for a given (observer, video) pair, is first computed for each step (segment) *t* of the discretised signal of length *T*, $1\le t\le T$, including all EEG channels. Then, the pairwise distances $d^{(t)}_{ij}=d(\textbf{f}_t^{(i)}, \textbf{f}_t^{(j)})$ between participants *i* and *j* watching the same video stimuli are computed, and Eq. 2 can therefore be evaluated.

Compared to the GLIMPSE measure, which is computed on gaze data, there are two differences with the EEG signals. On the one hand, while gaze is simply 2D data (gaze coordinates), we have *k*-dimensional features (not simply two). On the other hand, and most importantly, the gaze coordinates were bounded by the display size, whereas EEG features values are unbounded. This affects our consistency computation in several ways: how to scale/normalise the data, how to compute the distances, and how to select the threshold.

As in the visual salience case, the temporal parameter of the gaze-based consistency measure is omitted for the sake of simplicity. Averaging the feature vectors of neighbouring temporal steps or smoothing the resulting signal $s_B(t)$ would capture the temporal effect. In our case, since the EEG features are computed from time windows of the EEG signals, the temporal smoothing effect is already implicitly considered.

The cosine distance was used for pairwise distance computations:

4 $$\begin{aligned} d(\textbf{f}_t^{(i)}, \textbf{f}_t^{(j)}) = 1 - \frac{\textbf{f}_t^{(i)} \cdot \textbf{f}_t^{(j)}}{||\textbf{f}_t^{(i)}|| \cdot ||\textbf{f}_t^{(j)}||}, \end{aligned}$$

since being bounded to $[-1,1]$, a threshold for $\theta _s$ is easier to set, and feature normalisation is not required.

For the feature vectors of the EEG signal $\textbf{f}$, we used the hand-crafted features as computed in [19] for supervised classification of affective states. Five frequency bands were considered: Delta (0.5–4 Hz), Theta (4–7 Hz), Alpha (8–12 Hz), Beta (13–30 Hz), and Gamma (31+ Hz), and 5 features were computed per band and EEG channel: three Hjorth parameters (activity, mobility and complexity), spectral entropy, and signal energy, resulting in 800 (5 bands × 5 features × 32 EEG channels) features. Note that these features are one reasonable choice, among many possibly others, to represent the EEG signals since our method relies on inter-subject brain consistency for their potential to predict temporal visual attention.

### Measuring correlation

Given $s_V(t)$ computed on visual information (here, salience maps) and $s_B(t)$ computed on brain data (here, EEG features), we want to quantify how they relate over time $t\in [1,T]$. Fully global metrics such as the Euclidean distance are not adequate in capturing local similarities. To overcome this limitation, we propose a local-based comparison with the overall idea of finding in how many temporally local segments the compared signals are similar enough.

#### Comparison method

For our comparison accounting for local similarities, we introduce the following notation:

*Segment length* ($l$). Signals will be compared using temporally aligned segments of $s_V$ and $s_B$, each of length $l$.

*Comparison criterion* ($c$). Each segment pair will be compared using a particular success criteria $c$ which, in turn, relies on a metric and a threshold:

- *Comparison metric* ($\sigma$). The underlying metric used as part of the comparison criterion.
- *Comparison threshold* ($\theta _c$). A threshold on the metric decides whether the compared segments meet the “close enough” criterion for the corresponding metric.

Formally, let $s^{(k)}_V$ and $s^{(k)}_B$ be two aligned segments each of length $l$ from the compared signals of total length *T*, $1\le l\le T$. Then, whether these segments are “close enough” is evaluated with a criterion $c$ as a function of a threshold $\theta _c$, $c(s^{(k)}_V,s^{(k)}_B;\theta _c)$. Next, the final measure of correlation, $\rho$, is computed as the ratio of “matching” tests:

5 $$\begin{aligned} \rho (s_V,s_B) = \frac{1}{M} \sum _{k=1}^{M} \mathbbm {1}[c(s_V^{(k)},s_B^{(k)};\theta _c)], \end{aligned}$$

where $s_V^{(k)}$ and $s_B^{(k)}$ are the temporally aligned *k*-th segment of $s_V$ and $s_B$. Depending on the underlying metric being used, the criteria $c$ and number of tests $M$ differ, as follows.

Let $s_V^{t:t+l}$ and $s_B^{t:t+l}$ be the compared segments, each of length *l*. For element-wise metrics (such as intersection over union, IoU), the criterion is met if the metric applies to *all* values of the compared segments of length $l$, i.e.

$$\begin{aligned} c(s_V^{t:t+l},s_B^{t:t+l};\theta _c)= & \textsf {True}\quad \text { if}\; \sigma (s_V^{i},s_B^{i})<\theta _c\quad \\ & \forall \; i\in \{t,t+1,\ldots ,t+l-1\}. \end{aligned}$$

In this case, $M$ is the total number of possible overlapping segments of length $l$.

Correlation-based metrics (such as Spearman correlation), however, cannot be evaluated element-wise, since they require a set of values. In this case, the criteria is met if the metric is higher than a threshold for the time-sorted values within a segment of length $l\ge 2$ namely,

$$\begin{aligned} c(s_V^{t:t+l},s_B^{t:t+l};\theta _c) =\sigma (s_V^{t:t+l},s_B^{t:t+l})>\theta _c. \end{aligned}$$

If $c(s_V^{t:t+l},s_B^{t:t+l};\theta _c)$ is ${\textsf {True}}$, then all positions of the signals corresponding to the evaluated segment (i.e. {$t, t+1, \ldots , t+l-1$}) are marked as ${\textsf {True}}$, and therefore *M* equals the length *T* of the full signal.

#### Some practical considerations

Before computing $\rho$, the signals are smoothed for noise reduction, which is particularly important for some comparison criteria (e.g. derivative-based ones). The proposed comparison method is general enough to accommodate a range of metrics and comparison properties (e.g. global vs local, strict vs lenient). Possible comparison criteria $c$ are Spearman, IoU, absolute difference, and derivative-based (sign). For the sign criterion, $\theta _c=0$ if we only wish to test for equal derivative sign (i.e both increasing or decreasing simultaneously). In the following, we will consider the Spearman correlation, whose values range in $[-1,1]$ and have easy interpretation: positive (respectively, negative) correlations range from 0 (no correlation) to 1 (maximum positive correlation) or, respectively, $-1$ (maximum negative correlation). Being a rank-based metric, Spearman correlation is a good choice because it is insensitive to the particular values of the scores compared scores and relies on the correlation of their relative values.

The segment length $l$ and the threshold $\theta _c$ can regulate how demanding the comparison can be. The longer the segment and/or the higher the threshold, the stricter the comparison. Note that $l$ defines how local or global the comparison is, with low values for more local and high values for more global. An example of the effect of the segment length $l$ and the comparison threshold $\theta _c$ on the final correlation $\rho$ is given in Sect. 3.2.2.

#### Relevance of correlation

To understand whether the correlation $\rho$ between $s_V$ and $s_B$ is relevant (i.e. meaningful or significant), we consider a random baseline as a reference. Formally, we compare $\rho = \rho (s_V,s_B)$ with $\tilde{\rho }=\rho (s_V,\tilde{s}_B)$, where $\tilde{s}_B$ is a *perturbed* version of the signal $s_B$. Therefore, if $\rho$ is higher than the baseline $\tilde{\rho }$, it is an evidence of the relevance of the correlation. The considered perturbed signal $\tilde{s}_B$ is a random permutation of $s_B$, i.e. a temporal shuffling, thus preserving the distribution of values of $s_B$ but randomising their order.

To quantify the difference between $\rho$ and $\tilde{\rho }$, we use Cohen’s *d* [29], which will therefore represents the effect size [30],

6 $$\begin{aligned} d = \frac{\rho _m -\tilde{\rho }_m}{s_p}, \end{aligned}$$

where the subscript *m* represents the mean of either $\rho$ and $\tilde{\rho }$ over the set of videos, and $s_p$ is the pooled standard deviation $s_p=(\rho _s^2 + \tilde{\rho }_s^2)/2$, with subscript *s* representing the standard deviation of the subscripted variable.

In the plots of results, we include the qualitative ranges for interpreting the numerical values proposed in [31] expanding the original Cohen’s ranges [29].

## Experimental work

### Datasets and additional details

The proposed methodology is tested on two datasets which include EEG data for a number of participants watching several videos. On the one hand, DEAP [32] includes EEG signals from 32 participants watching 40 video snippets. Our experiment is run over the 23 videos that were available from YouTube out of those 40. The features were computed on one-second-long segments for the 60-second length video snippet, resulting in $T=60$ timesteps. On the other hand, from MAHNOB [33] we used EEG signals of 27 participants and 17 videos. The videos were 60-second long, and the features were extracted on segments of length 2 s, resulting in $T=30$ timesteps.

For temporal smoothing of the $s_V$ and $s_B$ signals, a Gaussian filter with standard deviation 1 and 2, respectively, was applied to DEAP data, and a mean filter with window size 3 was applied to MAHNOB data. To account for the variability of the random permutations used to compute $\tilde{s}_B$, which results in different $\tilde{\rho }$, the reported effect sizes correspond to the average of 10 random permutations.

### Concept validation

#### Visual-based salience score

**Fig. 3:**
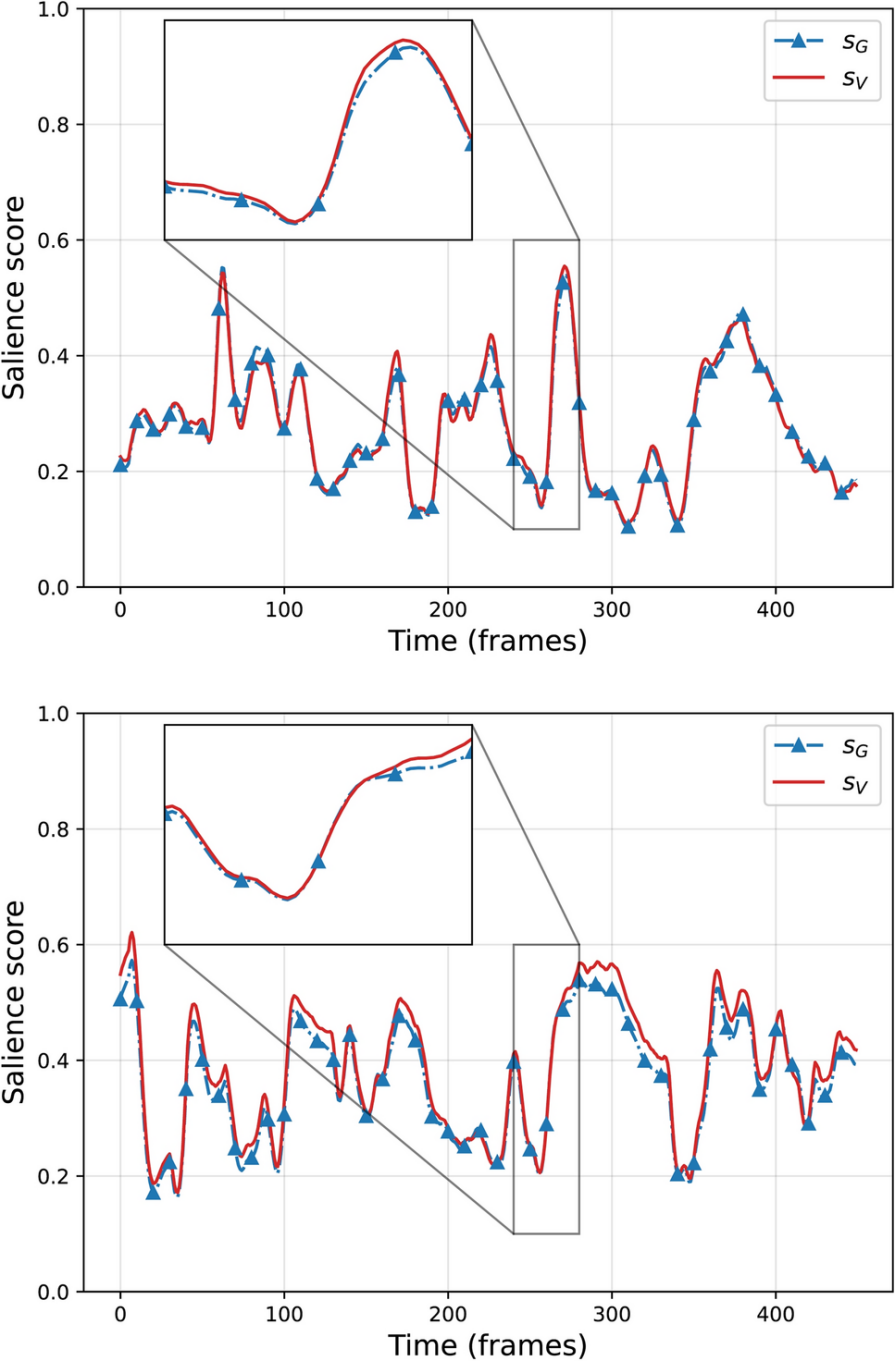
Comparison of $s_V$ computed on gaze maps and $s_G$ on gaze points on two sample videos from SAVAM (Video 16, above; and Video 33, below). It can be observed how closely our definition of $s_V$ for 2D maps is to $s_G$ from a set of gaze points

As a sanity check, the proposed Eq. 3 was first tested on gaze maps from the SAVAM dataset [34], which has both gaze points and gaze-based salience maps. We verified that these scores highly correlate with the gaze-based definition (Fig. 3).

#### Correlation analysis

**Fig. 4:**
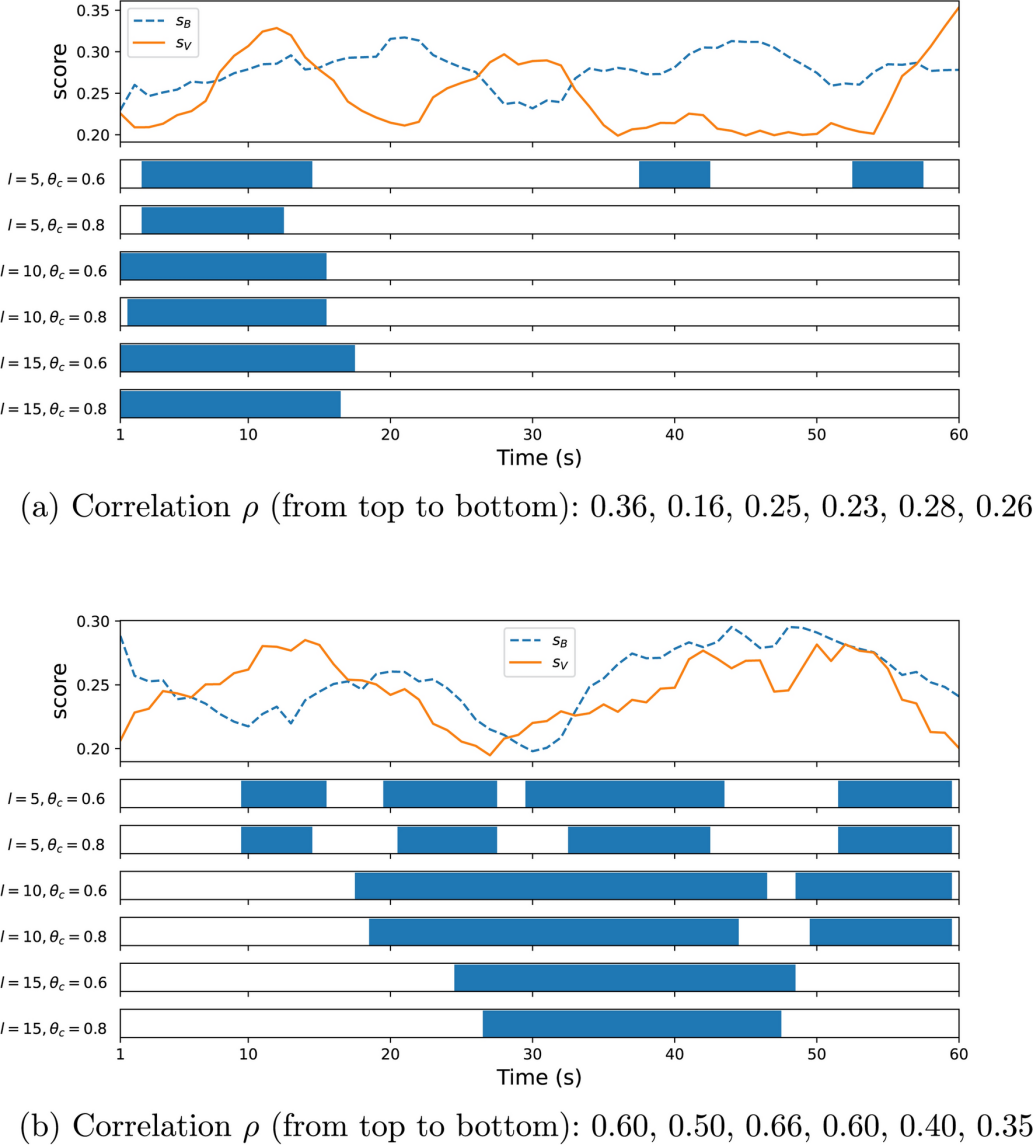
Examples of compared signals ($s_V$ and $s_B$) for two particular videos in DEAP (Video 1, top; Video 20, bottom) with varying correlation for different $l$ and $\theta _c$, and the corresponding $\rho$. For each pair $(l,\theta _c)$, the matching temporal segments using the comparison criterion $c$ (Sect. 2.4.1) are marked as blue bars when $c(t)$ is True

Some examples of compared signals ($s_V$ and $s_B$) and their corresponding $\rho$ are given in Fig. 4 for $l\in \{5,10,15\}$, which represents $8.3\%$, $16.7\%$ and $25\%$ of the signal length $T=60$ (seconds). It can be noticed how $\rho$ drops for increasing $\theta _c$ (for fixed $l$), and generally for increasing $l$. The matching temporal windows shrink when $\theta _c$ is increased for a given $l$, and the location of the matching windows may change with different values for $l$. The example Fig. 4b illustrates a case where the overall signals shapes matches most of the time, particularly in the second half minute.

### Results

**Fig. 5:**
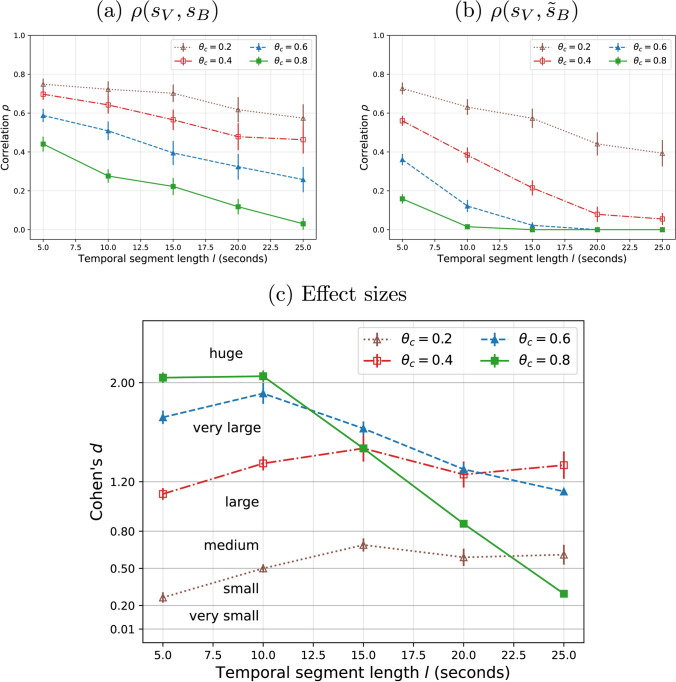
Correlation results with varying segment lengths and thresholds and comparison with the random (permutation) for DEAP.** a** Correlation between the original temporal salience scores;** b** correlation with perturbed temporal salience scores (from brain) and** c** Their effect sizes (Cohen’s *d*). Averages and standard error are reported for the set of videos (**a**,** b**) and for ten random permutations in each case (**c**)

**Fig. 6:**
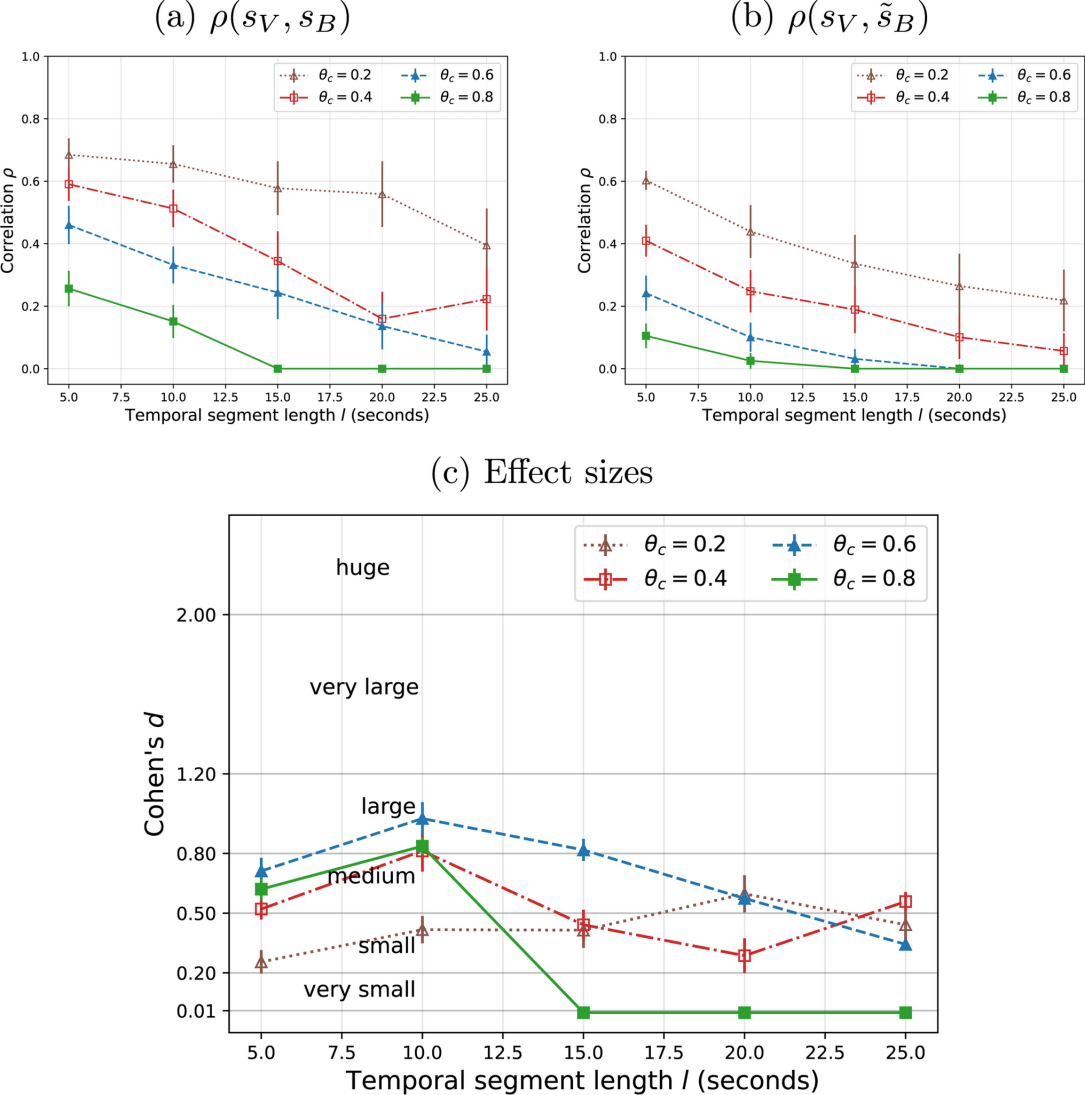
Correlation results with varying segment lengths and thresholds and comparison with the random (permutation) for MAHNOB. **a** Correlation between the original temporal salience scores; **b** correlation with perturbed temporal salience scores (from brain) and **c** their effect sizes (Cohen’s *d*). Averages and standard error are reported for the set of videos (**a**,** b**) and for ten random permutations in each case (**c**)

We compare all available videos and aggregate the performance metrics. For DEAP dataset, it can be seen that the mean correlation when the original signals are compared (Fig. 5a) is always higher than the baseline comparison (Fig. 5b). This suggests that, interestingly, inter-subject consistency of EEG signals can be an effective mechanism to predict temporal visual attention. As expected, the correlation decreases when either the segment length $l$ or the comparison threshold $\theta _c$ increase, because of the stricter comparison. Similar observations, but with smaller correlations can be observed for MAHNOB (Fig. 6a,b).

The effect size using Cohen’s *d* is observed to be mostly between medium and very large for DEAP (Fig. 5c) and between very small and large for MAHNOB (Fig. 6c), for the tested segment lengths and comparison thresholds, which confirms the differences observed in between $\rho$ and $\tilde{\rho }$. Understandably, the effect decreases with longer segment lengths $l$ and comparison thresholds $\theta _c$, since the correlation $\rho$ is lower and therefore gets closer to the baseline correlation $\tilde{\rho }$.

As an example of the influence of the parameters $(l, \theta _c)$, if we are too permissive and set $\theta _c$ to a low value (e.g. 0.2), then the correlation can be high even for the random baseline (Figs. 5b, 6b), resulting in low effect size (Figs. 5c, 6c). If we are too strict, correlation will be found to be low. Therefore, some tradeoff is generally required and, ultimately, the choice of $l$ and $\theta _c$ can be application-dependent.

### Additional analysis

To gain further insights, the analysis was repeated for different frequency bands, EEG features, EEG channel groups, different video subsets according to their emotional annotations, and for automatically selected features.

#### Frequency bands

For the $$(l,\theta _c)$$ pairs with the best performance $$(\rho >0.4$$ and Cohen’s $$d>0.5)$$ we repeated the analysis for each of the frequency bands (Table 1). The row-wise maximum mean value is bold-faced in this and the following tables.  The results with the Delta band are in most cases better than with any of the other bands and than all of them joined. This may look surprising since the Delta band is typically associated to deep sleep stages. However, in the context of our crowdsourcing-based method, it is simply that this band turns out to help in quantifying the inter-subject brain consistency in agreement with temporal visual attention. In other words, the brain responses of the participants are more aptly aligned when using features extracted from this band.

**Table 1:** Results (Cohen’s *d*, ${\text {mean}}{\,[{\text {standard error}}]}$) per frequency band

$l$	$\theta _c$	All	Delta	Theta	Alpha	Beta	Gamma
*DEAP*							
5	0.4	${{\textbf {1}}.{\textbf {10}}}{\,[{0.05}]}$	${{0.88}}{\,[{0.04}]}$	${{0.90}}{\,[{0.06}]}$	${{0.82}}{\,[{0.08}]}$	${{1.06}}{\,[{0.06}]}$	${{0.78}}{\,[{0.06}]}$
5	0.6	${{1.72}}{\,[{0.05}]}$	${{\textbf {2}}.{\textbf {15}}}{\,[{0.08}]}$	${{1.60}}{\,[{0.07}]}$	${{1.61}}{\,[{0.07}]}$	${{1.65}}{\,[{0.09}]}$	${{1.54}}{\,[{0.06}]}$
5	0.8	${{2.04}}{\,[{0.04}]}$	${{\textbf {3}}.{\textbf {28}}}{\,[{0.10}]}$	${{1.91}}{\,[{0.08}]}$	${{1.78}}{\,[{0.06}]}$	${{2.14}}{\,[{0.08}]}$	${{2.03}}{\,[{0.09}]}$
10	0.4	${{1.35}}{\,[{0.06}]}$	${{\textbf {1}}.{\textbf {60}}}{\,[{0.08}]}$	${{0.82}}{\,[{0.04}]}$	${{0.94}}{\,[{0.05}]}$	${{1.04}}{\,[{0.09}]}$	${{0.92}}{\,[{0.07}]}$
10	0.6	${{1.91}}{\,[{0.08}]}$	${{\textbf {2}}.{\textbf {57}}}{\,[{0.10}]}$	${{1.61}}{\,[{0.09}]}$	${{1.67}}{\,[{0.05}]}$	${{1.75}}{\,[{0.07}]}$	${{1.43}}{\,[{0.06}]}$
15	0.2	${{0.69}}{\,[{0.05}]}$	${{\textbf {0}}.{\textbf {73}}}{\,[{0.06}]}$	${{0.65}}{\,[{0.04}]}$	${{0.57}}{\,[{0.08}]}$	${{0.70}}{\,[{0.08}]}$	${{0.22}}{\,[{0.04}]}$
15	0.4	${{1.47}}{\,[{0.11}]}$	${{\textbf {1}}.{\textbf {89}}}{\,[{0.07}]}$	${{1.39}}{\,[{0.04}]}$	${{1.53}}{\,[{0.05}]}$	${{1.14}}{\,[{0.08}]}$	${{1.03}}{\,[{0.07}]}$
20	0.2	${{0.59}}{\,[{0.07}]}$	${{0.80}}{\,[{0.04}]}$	${{\textbf {0}}.{\textbf {81}}}{\,[{0.02}]}$	${{0.65}}{\,[{0.06}]}$	${{0.60}}{\,[{0.07}]}$	${{0.15}}{\,[{0.05}]}$
20	0.4	${{1.26}}{\,[{0.10}]}$	${{\textbf {1}}.{\textbf {95}}}{\,[{0.09}]}$	${{1.42}}{\,[{0.05}]}$	${{1.24}}{\,[{0.05}]}$	${{1.14}}{\,[{0.09}]}$	${{0.93}}{\,[{0.06}]}$
25	0.2	${{0.61}}{\,[{0.08}]}$	${{0.94}}{\,[{0.05}]}$	${{\textbf {1}}.{\textbf {05}}}{\,[{0.06}]}$	${{0.59}}{\,[{0.06}]}$	${{0.41}}{\,[{0.07}]}$	${{0.24}}{\,[{0.04}]}$
25	0.4	${{1.33}}{\,[{0.11}]}$	${{\textbf {1}}.{\textbf {37}}}{\,[{0.06}]}$	${{1.02}}{\,[{0.07}]}$	${{0.96}}{\,[{0.05}]}$	${{0.96}}{\,[{0.05}]}$	${{0.91}}{\,[{0.06}]}$
* MAHNOB*							
5	0.4	${{0.52}}{\,[{0.05}]}$	${{0.61}}{\,[{0.06}]}$	${{0.35}}{\,[{0.08}]}$	${{\textbf {0}}.{\textbf {62}}}{\,[{0.08}]}$	${{0.51}}{\,[{0.05}]}$	${{0.51}}{\,[{0.07}]}$
5	0.6	${{0.71}}{\,[{0.07}]}$	${{\textbf {0}}.{\textbf {97}}}{\,[{0.06}]}$	${{0.36}}{\,[{0.06}]}$	${{0.31}}{\,[{0.09}]}$	${{0.72}}{\,[{0.05}]}$	${{0.79}}{\,[{0.07}]}$
10	0.4	${{\textbf {0}}.{\textbf {81}}}{\,[{0.10}]}$	${{0.81}}{\,[{0.07}]}$	${{0.59}}{\,[{0.08}]}$	${{0.51}}{\,[{0.09}]}$	${{0.52}}{\,[{0.06}]}$	${{0.58}}{\,[{0.09}]}$
20	0.2	${{\textbf {0}}.{\textbf {60}}}{\,[{0.09}]}$	${{0.27}}{\,[{0.07}]}$	${{0.07}}{\,[{0.12}]}$	${{0.46}}{\,[{0.11}]}$	${{0.32}}{\,[{0.07}]}$	${{0.17}}{\,[{0.11}]}$

#### Features

Similarly, we repeated the analysis for each feature. Two features (Hjorth mobility and spectral entropy) did not produce correlation and results include the other three (Hjorth activity, Hjorth complexity and Energy). According to the results (Table 2) some feature may produce better performance for some particular combination of the parameters $(l,\,\theta _{c} ),$ but there is no feature that is generally preferable.

**Table 2:** Results (Cohen’s *d*, ${\text {mean}}{\,[{\text {standard error}}]}$) per feature

$i$	$\theta _c$	All	Hjorth activity	Hjorth complexity	Energy
* DEAP*					
5	0.4	${{1.10}}{\,[{0.05}]}$	${{1.13}}{\,[{0.07}]}$	${{1.11}}{\,[{0.04}]}$	${{\textbf {1}}.{\textbf {18}}}{\,[{0.07}]}$
5	0.6	${{\textbf {1}}.{\textbf {72}}}{\,[{0.05}]}$	${{1.62}}{\,[{0.07}]}$	${{1.71}}{\,[{0.06}]}$	${{1.66}}{\,[{0.07}]}$
5	0.8	${{2.04}}{\,[{0.04}]}$	${{\textbf {2}}.{\textbf {18}}}{\,[{0.07}]}$	${{2.09}}{\,[{0.04}]}$	${{2.17}}{\,[{0.06}]}$
10	0.4	${{\textbf {1}}.{\textbf {35}}}{\,[{0.06}]}$	${{1.13}}{\,[{0.06}]}$	${{1.35}}{\,[{0.06}]}$	${{1.20}}{\,[{0.06}]}$
10	0.6	${{\textbf {1}}.{\textbf {91}}}{\,[{0.08}]}$	${{1.83}}{\,[{0.09}]}$	${{1.91}}{\,[{0.08}]}$	${{1.83}}{\,[{0.09}]}$
15	0.2	${{0.69}}{\,[{0.05}]}$	${{0.68}}{\,[{0.07}]}$	${{\textbf {0}}.{\textbf {73}}}{\,[{0.06}]}$	${{0.68}}{\,[{0.07}]}$
15	0.4	${{1.47}}{\,[{0.11}]}$	${{1.56}}{\,[{0.07}]}$	${{1.46}}{\,[{0.11}]}$	${{\textbf {1}}.{\textbf {59}}}{\,[{0.08}]}$
20	0.2	${{0.59}}{\,[{0.07}]}$	${{1.04}}{\,[{0.06}]}$	${{0.58}}{\,[{0.07}]}$	${{\textbf {1}}.{\textbf {09}}}{\,[{0.06}]}$
20	0.4	${{1.26}}{\,[{0.10}]}$	${{1.40}}{\,[{0.06}]}$	${{1.25}}{\,[{0.10}]}$	${{\textbf {1}}.{\textbf {40}}}{\,[{0.06}]}$
25	0.2	${{0.61}}{\,[{0.08}]}$	${{\textbf {0}}.{\textbf {98}}}{\,[{0.09}]}$	${{0.60}}{\,[{0.08}]}$	${{0.97}}{\,[{0.08}]}$
25	0.4	${{\textbf {1}}.{\textbf {33}}}{\,[{0.11}]}$	${{1.12}}{\,[{0.06}]}$	${{1.33}}{\,[{0.10}]}$	${{1.10}}{\,[{0.07}]}$
*MAHNOB*					
5	0.4	${{\textbf {0}}.{\textbf {52}}}{\,[{0.05}]}$	${{0.43}}{\,[{0.10}]}$	${{0.45}}{\,[{0.06}]}$	${{0.42}}{\,[{0.10}]}$
5	0.6	${{0.71}}{\,[{0.07}]}$	${{0.75}}{\,[{0.09}]}$	${{\textbf {0}}.{\textbf {79}}}{\,[{0.08}]}$	${{0.74}}{\,[{0.10}]}$
10	0.4	${{0.81}}{\,[{0.10}]}$	${{0.32}}{\,[{0.08}]}$	${{\textbf {0}}.{\textbf {84}}}{\,[{0.10}]}$	${{0.39}}{\,[{0.08}]}$
20	0.2	${{\textbf {0}}.{\textbf {60}}}{\,[{0.09}]}$	${{0.03}}{\,[{0.07}]}$	${{0.54}}{\,[{0.09}]}$	${{0.05}}{\,[{0.07}]}$

#### Channel groups

**Fig. 7:**
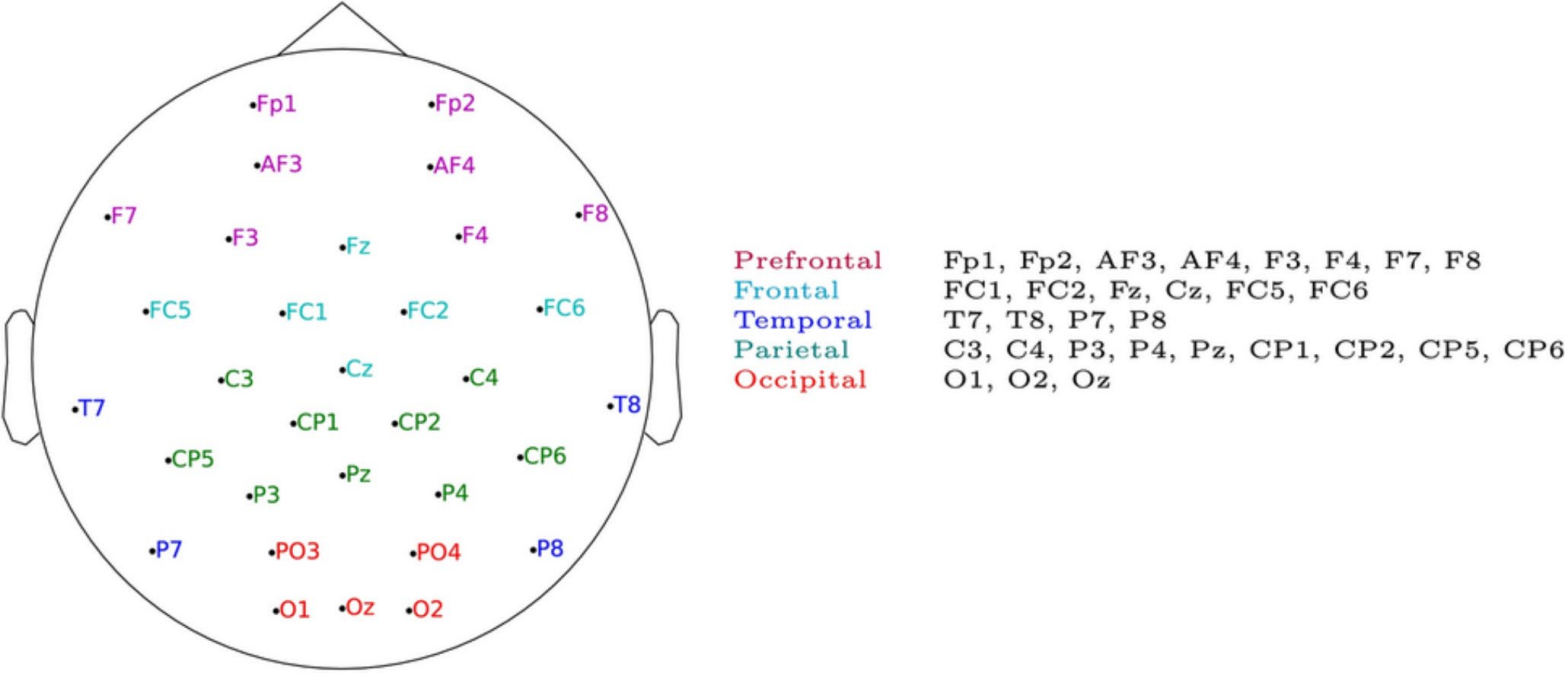
EEG channel groups considered. The occipital group did not include P03 and P04

The results per EEG channel groups (Fig. 7) are similar to the previous analysis in that there is no clear channel group that always gives the best performance (Table 3). Only in two combinations of the ($l$,$\theta _c$) parameters the occipital region (related to the visual cortex) provides higher performance. Similarly as discussed above, the explanation is not necessarily that the channels producing higher performance are more related to the visual attention, but that for some reason the inter-subject brain consistency using them turns out to be more useful for predicting the temporal visual salience.

**Table 3:** Results (Cohen’s *d*, ${\text {mean}}{\,[{\text {standard error}}]}$) per EEG channel group

$i$	$\theta _c$	All	Occipital	No occipital	Parietal	Temporal	Frontal	Prefrontal
*DEAP*								
5	0.4	${{1.10}}{\,[{0.05}]}$	${{\textbf {1}}.{\textbf {47}}}{\,[{0.07}]}$	${{0.84}}{\,[{0.04}]}$	${{1.18}}{\,[{0.05}]}$	${{1.46}}{\,[{0.05}]}$	${{1.01}}{\,[{0.05}]}$	${{1.25}}{\,[{0.06}]}$
5	0.6	${{1.72}}{\,[{0.05}]}$	${{2.16}}{\,[{0.05}]}$	${{1.58}}{\,[{0.04}]}$	${{1.90}}{\,[{0.06}]}$	${{\textbf {2}}.{\textbf {39}}}{\,[{0.08}]}$	${{1.70}}{\,[{0.07}]}$	${{1.87}}{\,[{0.08}]}$
5	0.8	${{2.04}}{\,[{0.04}]}$	${{\textbf {3}}.{\textbf {16}}}{\,[{0.07}]}$	${{2.42}}{\,[{0.06}]}$	${{2.68}}{\,[{0.10}]}$	${{2.70}}{\,[{0.08}]}$	${{2.41}}{\,[{0.09}]}$	${{2.43}}{\,[{0.08}]}$
10	0.4	${{1.35}}{\,[{0.06}]}$	${{1.44}}{\,[{0.07}]}$	${{1.22}}{\,[{0.05}]}$	${{\textbf {1}}.{\textbf {62}}}{\,[{0.10}]}$	${{1.31}}{\,[{0.07}]}$	${{1.09}}{\,[{0.08}]}$	${{1.34}}{\,[{0.06}]}$
10	0.6	${{1.91}}{\,[{0.08}]}$	${{2.08}}{\,[{0.09}]}$	${{1.84}}{\,[{0.10}]}$	${{\textbf {2}}.{\textbf {60}}}{\,[{0.13}]}$	${{1.78}}{\,[{0.07}]}$	${{1.69}}{\,[{0.06}]}$	${{1.77}}{\,[{0.07}]}$
15	0.2	${{0.69}}{\,[{0.05}]}$	${{0.61}}{\,[{0.06}]}$	${{0.58}}{\,[{0.05}]}$	${{0.57}}{\,[{0.10}]}$	${{\textbf {0}}.{\textbf {72}}}{\,[{0.06}]}$	${{0.52}}{\,[{0.06}]}$	${{0.63}}{\,[{0.06}]}$
15	0.4	${{1.47}}{\,[{0.11}]}$	${{1.49}}{\,[{0.07}]}$	${{1.34}}{\,[{0.08}]}$	${{1.42}}{\,[{0.09}]}$	${{1.33}}{\,[{0.05}]}$	${{1.12}}{\,[{0.07}]}$	${{\textbf {1}}.{\textbf {52}}}{\,[{0.09}]}$
20	0.2	${{0.59}}{\,[{0.07}]}$	${{0.58}}{\,[{0.06}]}$	${{0.48}}{\,[{0.06}]}$	${{\textbf {0}}.{\textbf {76}}}{\,[{0.07}]}$	${{0.67}}{\,[{0.08}]}$	${{0.40}}{\,[{0.08}]}$	${{0.52}}{\,[{0.06}]}$
20	0.4	${{1.26}}{\,[{0.10}]}$	${{1.36}}{\,[{0.06}]}$	${{1.12}}{\,[{0.08}]}$	${{\textbf {1}}.{\textbf {47}}}{\,[{0.07}]}$	${{1.24}}{\,[{0.09}]}$	${{1.01}}{\,[{0.06}]}$	${{1.22}}{\,[{0.08}]}$
25	0.2	${{0.61}}{\,[{0.08}]}$	${{0.88}}{\,[{0.06}]}$	${{0.54}}{\,[{0.08}]}$	${{\textbf {0}}.{\textbf {89}}}{\,[{0.09}]}$	${{0.81}}{\,[{0.04}]}$	${{0.40}}{\,[{0.07}]}$	${{0.54}}{\,[{0.08}]}$
25	0.4	${{\textbf {1}}.{\textbf {33}}}{\,[{0.11}]}$	${{1.11}}{\,[{0.05}]}$	${{1.17}}{\,[{0.11}]}$	${{1.01}}{\,[{0.08}]}$	${{1.11}}{\,[{0.08}]}$	${{1.03}}{\,[{0.06}]}$	${{1.03}}{\,[{0.07}]}$
* MAHNOB*								
5	0.4	${{0.52}}{\,[{0.05}]}$	${{0.46}}{\,[{0.05}]}$	${{0.42}}{\,[{0.04}]}$	${{0.63}}{\,[{0.08}]}$	${{0.67}}{\,[{0.07}]}$	${{\textbf {0}}.{\textbf {71}}}{\,[{0.10}]}$	${{0.41}}{\,[{0.07}]}$
5	0.6	${{0.71}}{\,[{0.07}]}$	${{0.62}}{\,[{0.08}]}$	${{0.54}}{\,[{0.05}]}$	${{0.80}}{\,[{0.06}]}$	${{\textbf {1}}.{\textbf {07}}}{\,[{0.07}]}$	${{0.90}}{\,[{0.07}]}$	${{0.63}}{\,[{0.06}]}$
10	0.4	${{0.81}}{\,[{0.10}]}$	${{0.34}}{\,[{0.08}]}$	${{0.67}}{\,[{0.11}]}$	${{0.40}}{\,[{0.08}]}$	${{0.84}}{\,[{0.09}]}$	${{\textbf {0}}.{\textbf {86}}}{\,[{0.05}]}$	${{0.70}}{\,[{0.12}]}$
20	0.2	${{\textbf {0}}.{\textbf {60}}}{\,[{0.09}]}$	${{0.12}}{\,[{0.07}]}$	${{0.43}}{\,[{0.10}]}$	${{0.19}}{\,[{0.08}]}$	${{0.53}}{\,[{0.08}]}$	${{0.41}}{\,[{0.08}]}$	${{0.34}}{\,[{0.10}]}$

#### Emotional dimensions

To look into the effect of the emotional contents of the video stimuli, we split the analysis into subsets of videos according to the different combinations of low/high/any (L, H, *) valence (V) and arousal (A). The results (Table 4) suggest that there are differences in the correlation depending on the emotional dimensions. An interesting observation is that in 8 out of 11 $(l, \theta _c)$ parameter combinations for DEAP, the biggest correlation happens in high-arousal conditions (either LVHA or *VHA), which suggests that for high-arousal contents, the agreement in the brain responses from multiple participants correlates better with the visual consistence salience score. This observation may be related with the known fact that high-arousal emotions are easier to decode [35].

**Table 4:** Results (Cohen’s *d*, ${\text {mean}}{\,[{\text {standard error}}]}$) per video groups according to their emotional dimensions

(a) DEAP										
$i$	$\theta _c$	All videos	LVLA	LVHA	HVLA	HVHA	LV*A	HV*A	*VLA	*VHA
5	0.4	${{1.10}}{\,[{0.05}]}$	${{0.83}}{\,[{0.05}]}$	${{0.75}}{\,[{0.05}]}$	${{0.78}}{\,[{0.09}]}$	${{0.72}}{\,[{0.03}]}$	${{1.04}}{\,[{0.07}]}$	${{1.19}}{\,[{0.06}]}$	${{0.78}}{\,[{0.11}]}$	${{\textbf {1}}.{\textbf {26}}}{\,[{0.08}]}$
5	0.6	${{1.72}}{\,[{0.05}]}$	${{1.68}}{\,[{0.07}]}$	${{1.49}}{\,[{0.05}]}$	${{1.58}}{\,[{0.08}]}$	${{1.20}}{\,[{0.05}]}$	${{2.04}}{\,[{0.06}]}$	${{1.92}}{\,[{0.05}]}$	${{1.47}}{\,[{0.09}]}$	${{\textbf {2}}.{\textbf {16}}}{\,[{0.10}]}$
5	0.8	${{2.04}}{\,[{0.04}]}$	${{2.17}}{\,[{0.05}]}$	${{2.46}}{\,[{0.04}]}$	${{2.27}}{\,[{0.11}]}$	${{2.16}}{\,[{0.07}]}$	${{2.45}}{\,[{0.05}]}$	${{2.39}}{\,[{0.05}]}$	${{2.17}}{\,[{0.09}]}$	${{\textbf {2}}.{\textbf {51}}}{\,[{0.06}]}$
10	0.4	${{1.35}}{\,[{0.06}]}$	${{0.87}}{\,[{0.06}]}$	${{1.18}}{\,[{0.06}]}$	${{1.29}}{\,[{0.12}]}$	${{0.88}}{\,[{0.04}]}$	${{1.18}}{\,[{0.08}]}$	${{\textbf {1}}.{\textbf {67}}}{\,[{0.09}]}$	${{0.84}}{\,[{0.06}]}$	${{1.21}}{\,[{0.06}]}$
10	0.6	${{1.91}}{\,[{0.08}]}$	${{1.39}}{\,[{0.07}]}$	${{\textbf {2}}.{\textbf {29}}}{\,[{0.09}]}$	${{1.66}}{\,[{0.11}]}$	${{1.36}}{\,[{0.07}]}$	${{1.83}}{\,[{0.04}]}$	${{1.83}}{\,[{0.09}]}$	${{1.22}}{\,[{0.05}]}$	${{2.15}}{\,[{0.09}]}$
15	0.2	${{0.69}}{\,[{0.05}]}$	${{0.32}}{\,[{0.07}]}$	${{0.77}}{\,[{0.06}]}$	${{0.63}}{\,[{0.13}]}$	${{0.20}}{\,[{0.07}]}$	${{\textbf {0}}.{\textbf {79}}}{\,[{0.05}]}$	${{0.53}}{\,[{0.07}]}$	${{0.21}}{\,[{0.05}]}$	${{0.44}}{\,[{0.07}]}$
15	0.4	${{1.47}}{\,[{0.11}]}$	${{0.92}}{\,[{0.07}]}$	${{\textbf {1}}.{\textbf {77}}}{\,[{0.03}]}$	${{0.82}}{\,[{0.13}]}$	${{0.92}}{\,[{0.06}]}$	${{1.31}}{\,[{0.04}]}$	${{1.03}}{\,[{0.10}]}$	${{0.60}}{\,[{0.05}]}$	${{1.38}}{\,[{0.09}]}$
20	0.2	${{0.59}}{\,[{0.07}]}$	${{0.17}}{\,[{0.07}]}$	${{\textbf {0}}.{\textbf {81}}}{\,[{0.05}]}$	${{0.43}}{\,[{0.11}]}$	${{0.39}}{\,[{0.07}]}$	${{0.76}}{\,[{0.05}]}$	${{0.68}}{\,[{0.10}]}$	${{-0.04}}{\,[{0.06}]}$	${{0.61}}{\,[{0.07}]}$
20	0.4	${{1.26}}{\,[{0.10}]}$	${{0.81}}{\,[{0.08}]}$	${{\textbf {1}}.{\textbf {72}}}{\,[{0.08}]}$	${{0.71}}{\,[{0.12}]}$	${{1.20}}{\,[{0.05}]}$	${{1.09}}{\,[{0.06}]}$	${{1.21}}{\,[{0.09}]}$	${{0.60}}{\,[{0.06}]}$	${{1.42}}{\,[{0.07}]}$
25	0.2	${{0.61}}{\,[{0.08}]}$	${{0.34}}{\,[{0.08}]}$	${{0.72}}{\,[{0.04}]}$	${{0.39}}{\,[{0.11}]}$	${{0.57}}{\,[{0.08}]}$	${{\textbf {0}}.{\textbf {84}}}{\,[{0.06}]}$	${{0.68}}{\,[{0.10}]}$	${{0.09}}{\,[{0.07}]}$	${{0.78}}{\,[{0.08}]}$
25	0.4	${{1.33}}{\,[{0.11}]}$	${{1.14}}{\,[{0.10}]}$	${{1.04}}{\,[{0.05}]}$	${{1.10}}{\,[{0.09}]}$	${{1.07}}{\,[{0.07}]}$	${{1.00}}{\,[{0.06}]}$	${{1.12}}{\,[{0.09}]}$	${{0.64}}{\,[{0.05}]}$	${{\textbf {1}}.{\textbf {69}}}{\,[{0.08}]}$

(b) MAHNOB										
$l$	$\theta _c$	All	LVLA	LVHA	HVLA	HVHA	LV*A	HV*A	*VLA	*VHA
5	0.4	${{0.52}}{\,[{0.05}]}$	${{0.52}}{\,[{0.05}]}$	${{0.47}}{\,[{0.06}]}$	${{\textbf {0}}.{\textbf {80}}}{\,[{0.15}]}$	${{0.70}}{\,[{0.12}]}$	${{0.30}}{\,[{0.07}]}$	${{0.36}}{\,[{0.09}]}$	${{0.30}}{\,[{0.08}]}$	${{0.42}}{\,[{0.05}]}$
5	0.6	${{0.71}}{\,[{0.07}]}$	${{0.67}}{\,[{0.07}]}$	${{0.84}}{\,[{0.07}]}$	${{\textbf {1}}.{\textbf {17}}}{\,[{0.14}]}$	${{0.80}}{\,[{0.07}]}$	${{0.56}}{\,[{0.07}]}$	${{0.60}}{\,[{0.11}]}$	${{0.50}}{\,[{0.09}]}$	${{0.62}}{\,[{0.09}]}$
10	0.4	${{0.81}}{\,[{0.10}]}$	${{0.33}}{\,[{0.09}]}$	${{0.73}}{\,[{0.11}]}$	${{1.04}}{\,[{0.12}]}$	${{\textbf {1}}.{\textbf {58}}}{\,[{0.19}]}$	${{0.63}}{\,[{0.09}]}$	${{0.86}}{\,[{0.06}]}$	${{0.56}}{\,[{0.12}]}$	${{0.89}}{\,[{0.10}]}$
20	0.2	${{0.60}}{\,[{0.09}]}$	${{0.40}}{\,[{0.09}]}$	${{0.27}}{\,[{0.11}]}$	${{0.30}}{\,[{0.09}]}$	${{0.40}}{\,[{0.08}]}$	${{\textbf {0}}.{\textbf {41}}}{\,[{0.07}]}$	${{0.28}}{\,[{0.06}]}$	${{0.33}}{\,[{0.12}]}$	${{0.28}}{\,[{0.11}]}$

#### Feature selection

In order to identify whether some of the brain features are more useful than others for capturing correlations with the visual salience score, we use the LASSO (least absolute shrinkage and selection operator) regression analysis method [36] by solving the following optimisation problem:

7 $$\begin{aligned} \min _{\textbf{w}} \frac{1}{2N} ||\textbf{X}\textbf{w}- \textbf{y}||^2_2 + \lambda ||\textbf{w}||_1, \end{aligned}$$

which encourages sparsity in the used features via a regularisation term with the penalty factor $\lambda$ and $L_1$ prior, where *N* is the number of instances. In our case, $\textbf{X}$ is an $N\times M$ matrix with all the brain features ($M=800$) for each temporal segment for all videos, and $\textbf{y}$ is the $N\times 1$ vector of visual salience scores $s_V(t)$ for the corresponding segment *t*, and $\textbf{w}$ is the $M\times 1$ feature weights. More specifically, one row of $\textbf{X}$ corresponding to a temporal segment *t* is the sum over the feature vectors for the *K* participants, $\sum _{k=1}^{K} \textbf{f}_t^{(k)}$.

**Fig. 8:**
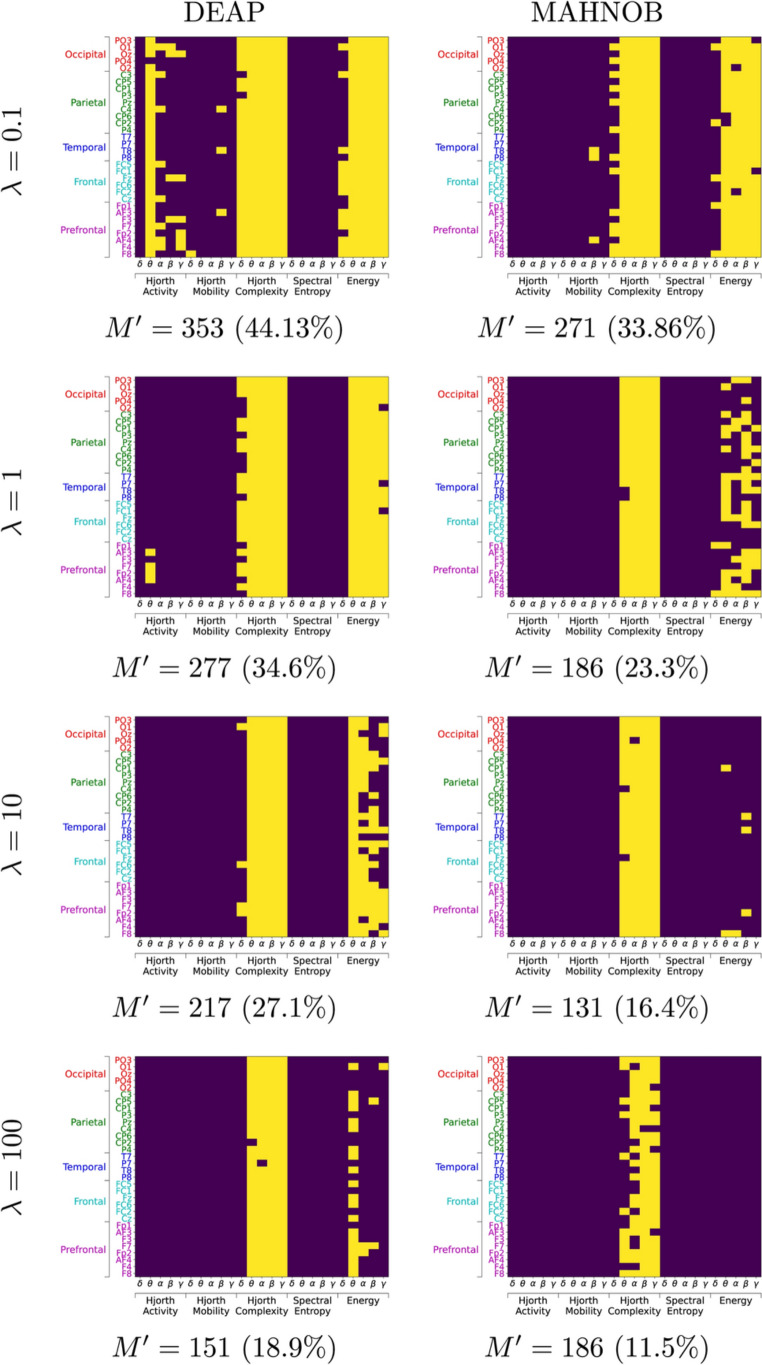
Selected features, marked in yellow corresponding to features *i* such that $\textbf{w}_i\ne 0$ found using Eq. 7 with $\lambda \in \{0.1,1,10,100\}$ (top to bottom) for DEAP (left) and MAHNOB (right). The number $M'$ of selected features and the percentage with respect to the original $M=800$ are given below each case. (color figure online)

Selected features (those corresponding to non-zero weights $\textbf{w}_i$) are shown in Fig. 8. According to this, the Hjorth complexity feature is important in nearly all bands (mostly for $\lambda =0.1$ and DEAP); and the other relevant feature is the energy for all but the Delta $(\delta)$band. Almost all channels are involved in those selected features. Note these results is in agreement with those in the per-feature analysis (Sect. 3.4), where no correlation was found when using Hjorth mobility or spectral entropy. When the sparsity factor is increased, and fewer features are selected, some energy features are dropped and the Hjorth complexity features are kept. This pattern is similar in DEAP and MAHNOB, with a difference being that the convenience of most channels holds for more cases in DEAP than in MAHNOB.

The correlation results using the reduced set of features (Table 5) are similar or slightly better than with the full set of features. This implies that a computational benefit might be expected by computing only the most relevant features (Fig. 8) or by performing feature selection and then proceeding with the reduced set.

**Table 5:** Results (Cohen’s *d*, ${\text {mean}}{\,[{\text {standard error}}]}$) with feature selection for different penalty factor $\lambda$

$l$	$\theta _c$	All features	$\lambda =0.1$	$\lambda =1$	$\lambda =10$	$\lambda =100$
* DEAP*						
5	0.4	${{1.10}}{\,[{0.05}]}$	${{1.10}}{\,[{0.05}]}$	${{1.10}}{\,[{0.05}]}$	${{1.10}}{\,[{0.05}]}$	${{\textbf {1}}.{\textbf {17}}}{\,[{0.05}]}$
5	0.6	${{\textbf {1}}.{\textbf {72}}}{\,[{0.05}]}$	${{1.72}}{\,[{0.05}]}$	${{1.72}}{\,[{0.05}]}$	${{1.72}}{\,[{0.05}]}$	${{1.67}}{\,[{0.06}]}$
5	0.8	${{2.04}}{\,[{0.04}]}$	${{2.04}}{\,[{0.04}]}$	${{2.04}}{\,[{0.04}]}$	${{2.04}}{\,[{0.04}]}$	${{\textbf {2}}.{\textbf {14}}}{\,[{0.04}]}$
10	0.4	${{\textbf {1}}.{\textbf {35}}}{\,[{0.06}]}$	${{1.35}}{\,[{0.06}]}$	${{1.35}}{\,[{0.06}]}$	${{1.35}}{\,[{0.06}]}$	${{1.29}}{\,[{0.06}]}$
10	0.6	${{\textbf {1}}.{\textbf {91}}}{\,[{0.08}]}$	${{1.91}}{\,[{0.08}]}$	${{1.91}}{\,[{0.08}]}$	${{1.91}}{\,[{0.08}]}$	${{1.90}}{\,[{0.10}]}$
15	0.2	${{\textbf {0}}.{\textbf {69}}}{\,[{0.05}]}$	${{0.69}}{\,[{0.05}]}$	${{0.69}}{\,[{0.05}]}$	${{0.69}}{\,[{0.05}]}$	${{0.68}}{\,[{0.05}]}$
15	0.4	${{1.47}}{\,[{0.11}]}$	${{1.47}}{\,[{0.11}]}$	${{1.47}}{\,[{0.11}]}$	${{1.47}}{\,[{0.11}]}$	${{\textbf {1}}.{\textbf {50}}}{\,[{0.12}]}$
20	0.2	${{0.59}}{\,[{0.07}]}$	${{0.59}}{\,[{0.07}]}$	${{0.59}}{\,[{0.07}]}$	${{0.59}}{\,[{0.07}]}$	${{\textbf {0}}.{\textbf {62}}}{\,[{0.08}]}$
20	0.4	${{1.26}}{\,[{0.10}]}$	${{1.26}}{\,[{0.10}]}$	${{1.26}}{\,[{0.10}]}$	${{1.26}}{\,[{0.10}]}$	${{\textbf {1}}.{\textbf {27}}}{\,[{0.10}]}$
25	0.2	${{\textbf {0}}.{\textbf {61}}}{\,[{0.08}]}$	${{0.61}}{\,[{0.08}]}$	${{0.61}}{\,[{0.08}]}$	${{0.61}}{\,[{0.08}]}$	${{0.56}}{\,[{0.07}]}$
25	0.4	${{1.33}}{\,[{0.11}]}$	${{1.33}}{\,[{0.11}]}$	${{1.33}}{\,[{0.11}]}$	${{1.33}}{\,[{0.11}]}$	${{\textbf {1}}.{\textbf {35}}}{\,[{0.11}]}$
* MAHNOB*						
5	0.4	${{\textbf {0}}.{\textbf {52}}}{\,[{0.05}]}$	${{0.48}}{\,[{0.05}]}$	${{0.47}}{\,[{0.06}]}$	${{0.40}}{\,[{0.06}]}$	${{0.47}}{\,[{0.04}]}$
5	0.6	${{0.71}}{\,[{0.07}]}$	${{0.70}}{\,[{0.07}]}$	${{0.68}}{\,[{0.07}]}$	${{0.72}}{\,[{0.06}]}$	${{\textbf {0}}.{\textbf {75}}}{\,[{0.07}]}$
10	0.4	${{0.81}}{\,[{0.10}]}$	${{0.80}}{\,[{0.10}]}$	${{0.76}}{\,[{0.10}]}$	${{\textbf {0}}.{\textbf {96}}}{\,[{0.10}]}$	${{0.84}}{\,[{0.10}]}$
20	0.2	${{\textbf {0}}.{\textbf {60}}}{\,[{0.09}]}$	${{0.59}}{\,[{0.09}]}$	${{0.56}}{\,[{0.09}]}$	${{0.42}}{\,[{0.07}]}$	${{0.23}}{\,[{0.07}]}$

## Discussion

The results of our study support the hypothesis that brain consistency across participants can be a marker of the temporal visual salience of the perceived dynamic visual contents. Understanding temporal visual attention and its connection to human cognition can offer insights into the research and practice in the Medical and HCI scopes. Some examples of potential applications include monitoring visual attention in educational settings (where learners watch video lectures, and trainers can get insights into the evolution of attention for either material re-design or instructional modifications), or in digital entertaining (e.g., finding whether contents in particular temporal segments draws the expected attention), etc. The multiple participants might be processing the visual dynamic contents at the same time (e.g., while watching a live event), which lends to an online analysis, or at different moments (e.g., different students process course materials at their convenience), which still allows an off-line analysis.

Our work is primarily exploratory in nature and the results suggest that a relevant correlation exists between temporal visual attention and crowdsourced brain signals. Certainly, a number of factors might affect the strength of this correlation and that our current model does not fully capture. Human variability and their EEG responses may hinder detecting and quantifying brain consistency. The visual contents can be arbitrarily complex and computational models of salience might not properly predict their visual salience. Visual attention is a complex cognitive process whose complete understanding and precise computational modelling is still imperfect. The temporal salience score relies on the salience maps, but other human factors and visual-contents aspects might be missing. For instance, people might be tired, or more or less emotionally engaged, and their reactions may consequently be different even for the same contents. The novelty and surprise aspects of the contents can also be relevant aspects, yet hard to account for.

In order to look into some of these open issues, future work might look into alternative brain and visual contents representations as well as visual-based and brain-based salience score computations. Visual contents other than videos, and cognitive tasks other than free viewing, might also be explored. The study of other temporal visual salience scores, and the role that alternative brain features may play in the context of this problem, is also relevant. Learning-based approaches can also be explored further; for instance, to map raw brain signals to temporal visual salience score. We observed that the effect size tended to peak at segment length of 10 seconds for both datasets, in particular for the stricter medium-large comparison thresholds $(\theta _c\in \{0.6,0.8\})$. It may be interesting to find out whether such a phenomenon is also consistent in other contexts.

## Conclusion

This work explores whether brain signals may reveal temporal visual attention implicitly encoded in the contents of dynamic visual stimuli such as videos. Our experiments provide preliminary evidence for this hypothesis. The proposed approach relies on the brain consistency concept using *brainsourcing* (i.e., crowdsourcing of brain signals).

The temporal visual attention is quantified by measuring how concentrated (vs. scattered) the salience maps of video frames are. Regarding EEG signals, a crowdsourcing approach measures how consistent the signals are per temporal segment among the set of participants who watched each video. A moderate (but much higher than random) locality-based correlation between the visual and brain agreement signals has been found. Under our experimental conditions, effect sizes using Cohen’s *d* turned out to be from very small to large in one dataset, and from medium to very large in another dataset. These findings are relevant due to its potential research and practical implications with brain-computer interfaces, e.g., for dynamic visual contents analysis, and in the medical domain.

## Appendix A: Implementation details

A naïve implementation of Eq. 3 is computationally prohibitive. We developed an efficient implementation which relies on two observations:

- The whole denominator of the equation can be factorised as $\sum _{i=1}^m \left\{ S(x_i,y_i) \cdot \left[ \sum _{j=1}^m S(x_j,y_j)\right] \right\}$ and, since the terms in the inner sum are the same as those in the outer sum, eventually the expression becomes $\left( \sum _{i=1}^m S(x_i,y_i)\right) ^2$. Thus, from an initially quadratic cost (naïve implementation) in the number of all image pixels $m$, $O(m^2)$, we arrive to a linear cost, $O(m)$ by simply squaring the sum of all values of the salience map.
- The computation of the numerator can be carried out through convolution operations with a filter consisting of a binary mask with ones on a centred circle of radius $\theta _s$. This convolution can be accelerated with a GPU and even computed in a single pass for a batch of salience maps (e.g. those corresponding to all the frames of a given video). If $C(x,y)$ is the output of the convolution, the final expression turns out to be $\sum _{i=1}^m S(x_i,y_i) \cdot C(x_i,y_i),$ i.e. a sum of the element-wise products of the salience map with its convoluted version, ending up again in a linear cost, $O(m)$, computation.

## Footnotes

1 Code at https://github.com/MichiganCOG/TASED-Net, model TASED_updated.pt.
